# Minimal change prion retinopathy: Morphometric comparison of retinal and brain prion deposits in Creutzfeldt-Jakob disease

**DOI:** 10.1016/j.exer.2022.109172

**Published:** 2022-07-06

**Authors:** Vanessa S. Goodwill, Ian Dryden, Jihee Choi, Chiara De Lillo, Katrin Soldau, Jorge Llibre-Guerra, Henry Sanchez, Christina J. Sigurdson, Jonathan H. Lin

**Affiliations:** a Department of Pathology, University of California, San Diego, CA, 92093, USA; b Departments of Pathology and Ophthalmology, Stanford University, CA, 94305, USA; c VA Palo Alto Healthcare System, Palo Alto, CA, 94304, USA; d Department of Neurology, University of California, San Francisco, CA, 94143, USA; e Department of Neurology, Washington University School of Medicine, St. Louis, MO, 63108, USA; f Global Brain Health Institute, University of California, San Francisco, CA, 94143, USA

**Keywords:** Prion, Retina, Brain, sporadic Creutzfeldt-Jakob disease, Spongiform encephalopathy, Immunohistochemistry, Eye

## Abstract

Sporadic Creutzfeldt-Jakob disease (sCJD) is the most commonly diagnosed human prion disease caused by the abnormal misfolding of the ‘cellular’ prion protein (PrP^C^) into the transmissible ‘scrapie-type’ prion form (PrP^Sc^). Neuropathologic evaluation of brains with sCJD reveals abnormal PrP^Sc^ deposits primarily in grey matter structures, often associated with micro-vacuolar spongiform changes in neuropil, neuronal loss, and gliosis. Abnormal PrP^Sc^ deposits have also been reported in the retina of patients with sCJD, but few studies have characterized the morphology of these retinal PrP^Sc^ deposits or evaluated for any retinal neurodegenerative changes. We performed histopathologic and morphometric analyses of retinal and brain prion deposits in 14 patients with sCJD. Interestingly, we discovered that the morphology of retinal PrP^Sc^ deposits generally differs from that of brain PrP^Sc^ deposits in terms of size and shape. We found that retinal PrP^Sc^ deposits consistently localize to the outer plexiform layer of the retina. Additionally, we observed that the retinal PrP^Sc^ deposits are not associated with the spongiform change, neuronal loss, and gliosis often seen in the brain. The stereotypic morphology and location of PrP^Sc^ deposits in sCJD retinas may help guide the use of ocular imaging devices in the detection of these deposits for a clinical diagnosis.

## 1. Introduction

Prion diseases are a heterogeneous class of rare fatal neurodegenerative diseases caused by the abnormal misfolding of the ‘cellular’ prion protein (PrP^C^) into the transmissible ‘scrapie-type’ prion form (PrP^Sc^) (Collinge, 2016; DeArmond and Prusiner, 1995; Halliday et al., 2014; Prusiner, 1991, 1998; Prusiner et al., 1990; Weissmann, 2004). The PrP^C^ peptide is ubiquitously expressed throughout the body and highly expressed within neurons via a single exon in the *PRNP* gene (Bendheim et al., 1992; DeArmond and Prusiner, 1995). Human prion diseases are generally classified into three groups: sporadic (85–90% idiopathic cases), genetic (10–15% inherited cases), and acquired (1–3% infectious cases) (Takada and Geschwind, 2013). Of the human prion diseases, sporadic Creutzfeldt-Jakob disease (sCJD) is the most commonly diagnosed, with a global incidence of 1–1.5 cases per 1 million population per year (Uttley et al., 2020). Although there are currently six major subtypes of sCJD with varying clinical and pathologic phenotypes (Parchi et al., 2009), patients typically present with rapidly progressive dementia associated with the accumulation of neurologic deficits, which culminate in death. Neuropathologic evaluations of brains with sCJD reveal abnormal PrP^Sc^ deposits in grey matter structures often associated with micro-vacuolar spongiform changes in the neuropil, neuronal loss, and gliosis.

Approximately 10–18% of patients with sCJD report visual symptoms upon initial presentation and about half of patients with sCJD experience visual symptoms at some point during their clinical course (Armstrong, 2006; Baiardi et al., 2016; Lueck et al., 2000; Rabinovici et al., 2006; Takada and Geschwind, 2013). Visual symptoms vary considerably in sCJD and can include blurred vision, nystagmus, diplopia, metamorphopsias, dyschromatopsia, supranuclear palsies, visuospatial disturbances, saccades, visual hallucinations, and anopsia (Cooper et al., 2005; Kropp et al., 1999; Lenk et al., 2018; Lueck et al., 2000; Ntantos et al., 2018; Proulx et al., 2008; Rabinovici et al., 2006; Will and Matthews, 1984; Wong et al., 2015). In the Heidenhain variant of sCJD, the visual symptoms are thought to arise from neurodegeneration of the visual occipital cortex (Baiardi et al., 2016). In other forms of sCJD, the neuroanatomic defects that underlie visual symptoms are less clear.

Electroretinographic b-wave abnormalities have been reported in patients with CJD (de Seze et al., 1998; Ishikawa et al., 2009). Histopathologic changes in CJD retinas have been variable. Several case reports of post-mortem CJD eyes described gliosis in the optic nerve and nerve fiber layer (NFL) and retinal ganglion cell (RGC) loss (Lesser et al., 1979; Roth et al., 1979; Sato et al., 1992; Tarkkanen and Haltia, 1980; Tsutsui et al., 1986); and two other studies also described spongiform changes in inner retinal structures (de Seze et al., 1998; Tsutsui et al., 1986). However, other CJD studies reported no structural abnormalities and no spongiform changes in the retina (Head et al., 2003; Roth et al., 1979; Tarkkanen and Haltia, 1980). With the advent of antibodies that detect PrP^Sc^ in tissue, PrP^Sc^ deposition has also been reported in retinas of patients with CJD (Head et al., 2003, 2005; Orru et al., 2018; Takao et al., 2018). Head and colleagues examined the eyes of two sCJD and two variant CJD (vCJD) autopsy cases and observed PrP^Sc^ deposition in the inner plexiform (IPL) and outer plexiform (OPL) layers of the retina (Head et al., 2003, 2005). Takao and colleagues reported similar findings in eyes from 16 CJD cases, which included sporadic, familial, and iatrogenic CJD forms (Takao et al., 2018). Orru and colleagues also observed PrP^Sc^ deposition in the OPL and to a lesser extent in the IPL in the eyes of 11 patients with sCJD (Orru et al., 2018). Together, these reports independently corroborate the deposition of PrP^Sc^ in the eyes of sCJD patients, most notably confined to the synaptic retinal laminae.

The aim of this present study is to expand upon previous research to provide a detailed characterization of the retinal pathology caused by PrP^Sc^ deposition in the eyes of patients with sCJD. We examined post-mortem eyes obtained from 14 cases of clinically and neuropathologically-documented sCJD, including the aforementioned 11 sCJD cases examined by Orru and colleagues (Orru et al., 2018). We compared the morphology of PrP^Sc^ deposits in the retina to those in the brain. We histologically evaluated the retina for evidence of spongiform change and neurodegeneration. Ultimately, we found intriguing differences in prion aggregate morphology, degree of spongiotic change, and neuronal loss in the eyes of decedents with sCJD compared to the brain.

**Fig. 1.**
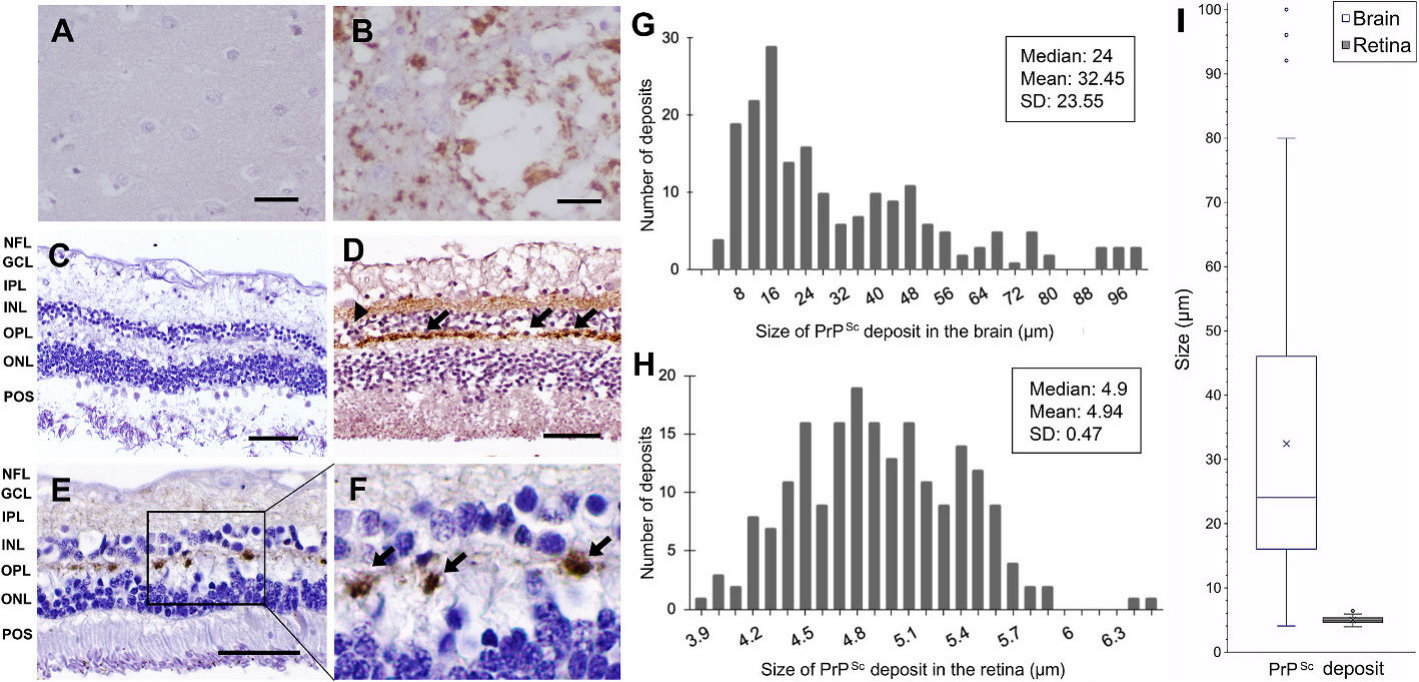
PrP^Sc^ immunohistochemistry (IHC) deposition in sCJD brain and retina. (A) Control brain cortex tissue shows no PrP^Sc^ immunolabeling. The scale bars in A and B represent 20 μm. (B) Perivacuolar depositions of PrP^Sc^ with extensive spongiotic neurodegeneration (vacuolation) in cerebral cortex section from a sCJD brain. (C) Control retina shows no PrP^Sc^ IHC staining. The scale bars in C, D, and E represent 50 μm. (D, E) PrP^Sc^ IHC staining of the retina in two different patients with sCJD. Immunolabeled linear ovoid deposits of PrP^Sc^ are shown in the OPL (arrows). In the IPL, granular (arrowhead) and synaptic-like deposits were seen in D and E, respectively. (F) Higher power of image E showing ovoid deposits in OPL (arrows). (G, H) Distribution of the size of PrP^Sc^ deposits in the brain (n = 195) and the retina (n = 202) in 14 patients with sCJD. (I) Box plot comparing the size of PrP^Sc^ deposits in the brain and retina. PrP^Sc^ deposits were significantly smaller in the retina than those in the brain (p < 0.001, Mann-Whitney *U* test and p < 0.001, Welch’s unpaired *t*-test). NFL. Nerve fiber layer, GCL. Ganglion cell layer, IPL. Inner plexiform layer, INL. Inner nuclear layer, OPL. Outer plexiform layer, ONL. Outer nuclear layer, POS. Photoreceptor outer segment.

From July 2015 to July 2017, eyes and brains were collected from 14 autopsies of clinically suspected sCJD at the UCSF Memory and Aging Center, 11 of which were previously described (Orru et al., 2018). Subjects consisted of four males and ten females with a mean age of 63 ± SD 8.96 (median 60, range 51–80) years old. The mean clinical disease course from symptom onset to death was 10.5 ± SD 8.38 (median 8, range 1.5–27) months, and 5 out of the 14 patients reported visual symptoms during the disease course (Table 1). Prion disease was confirmed in all cases by Western blot and immunohistochemistry (IHC) for PrP^Sc^ (Fig. 1B). Prion-related protein gene (*PRNP)* sequencing analysis showed no mutations in 13 cases, and one case was not sequenced. All three *PRNP* gene codon 129 polymorphisms were represented in the 13 sequenced cases: MM (n = 6), MV (n = 5), VV (n = 2).

Control cases consisted of eyes and brains from autopsies of five males and one female with a mean age of 70.3 ± SD 13.84 years (median 67.5, range 51–90) with no histories of retinal diseases or ocular surgeries except cataract surgeries (n = 3). The retinal histopathology in 4 of 6 control cases showed common age-related changes such as peripheral cystoid degeneration and drusen. The retinal histopathology in the remaining 2 control cases (3 and 6) was unremarkable. No PrP^Sc^ retinal deposits were found in any control retinas (Fig. 1C).

Two of the control patients had cognitive symptoms: control brain 4 showed “high” Alzheimer’s disease neuropathology by 2018 NIA-AA framework criteria (A2-Thal, B3-Braak, C2-CERAD); control brain 6 was sent to the National Prion Disease Surveillance Center and was negative for sCJD neuropathology. The remaining 4 control brains had no neurodegenerative disease neuropathology (Table 1). No prion deposits were found in any control brains by PrP^Sc^ IHC (Fig. 1A).

Formalin-fixed, paraffin-embedded brain and eye sections from sCJD and control cases were evaluated for further histologic and immunohistochemical PrP^Sc^ analysis.

Ocular tissues in fourteen patients with sCJD and six controls and brain tissues across the cerebral cortex, caudate, putamen, thalamus, and cerebellum in a case of sCJD (MV2; patient n° 4 in Table 1) were used for IHC staining and PrP^Sc^ quantification in this study. The eye tissues were formalin-fixed and immersed in 98% formic acid for 1 h. They were postfixed in formalin prior to paraffin embedding and sectioning. Sections of 4 μm thickness were placed onto positively charged silanized glass slides and deparaffinized. After being immersed in 96% formic acid for 5 min, the slides were washed with distilled water for 5 min and treated with 5 μg/ml of proteinase K for 7 min to remove natively expressed PrP^C^. Following another 5-min wash with distilled water, they were placed in citrate buffer and heated in a pressure cooker for 20 min for antigen retrieval. After being cooled and rinsed in distilled water the sections were incubated with anti-PrP Mab 12F10 (Cayman Chemical; 1:200 dilution) for 45 min, followed by anti-mouse IgG conjugated to biotin (Jackson Immunolabs; 1:250) and streptavidin-HRP (Jackson Immunolabs; 1:2000) for 30 min each. Labeling with diaminobenzidine (DAB) reagent (Thermo Fisher Scientific) was performed for brown chromogen visualization, and the sections were counterstained lightly with hematoxylin. PrP^Sc^ deposits within the eyes of sCJD patients were then evaluated and compared with normal controls and coarse/perivacuolar deposits within the brain.

Following preparation of autopsy brain and retina slides with prion IHC, 202 retinal deposits across 14 sCJD cases and 195 brain PrP^Sc^ deposits across the cerebral cortex and thalamus in a case of sCJD were photographed, and the greatest diameter of each PrP^Sc^ deposit was measured (Olympus CellSens Imaging Software, Waltham, MA).

The difference in the diameters of the PrP^Sc^ depositions in the brain and retina was statistically tested using the Mann-Whitney *U* test in SPSS. The difference in the mean diameters between brain and retina PrP^Sc^ deposits was statistically tested using Welch’s unpaired *t*-test. Standard deviations of the two groups were tested with the Brown-Forsythe test for their difference. P values of less than 0.05 were considered statistically significant.

In the 14 sCJD cases (Table 1) granular PrP^Sc^ deposition was found by IHC in several synaptic laminae of the retina (Fig. 1D and E). Similar to prior studies, PrP^Sc^ deposition was strongest in the OPL (14/14) (Fig. 1D and E). Fainter patchier PrP^Sc^ staining was also occasionally observed in the IPL in some cases (Fig. 1D and E). No consistent relationship between *PRNP* genotype and IHC staining was seen, although this is limited by our small sample size. No PrP^Sc^ deposition was seen in other retinal layers including retinal pigment epithelia (RPE), outer nuclear layer (ONL; rod and cone photoreceptor nuclei), inner nuclear layer (INL; retinal interneurons and Muller glia), ganglion cell layer (GCL), and nerve fiber layer (NFL). PrP^Sc^ deposits were not detected elsewhere in the eye including sclera, cornea, lens, choroid, and optic nerve. PrP^Sc^ deposition was completely absent in retinas of all 6 control cases (Fig. 1C).

Within the OPL, PrP^Sc^ strongly accumulated in discrete, ovoid deposits spaced along the length of the OPL, reminiscent in appearance to “beads-on-a-string” (Fig. 1D and E). These PrP^Sc^ deposits in the OPL also arose at fixed distances between the ONL and INL along the length of the lamina (Fig. 1D and E). Measurement of 202 ovoid PrP^Sc^ retinal deposits from the OPL showed an average greatest diameter of 4.94 μm with little variation (SD: ± 0.47 μm, Fig. 1H). In contrast to the stereotypic appearance of PrP^Sc^ deposits in the OPL of the retina, PrP^Sc^ deposits in the brain had highly irregular morphologies and widespread deposition (Fig. 1B). PrP^Sc^ deposits in the brain also had an average greatest diameter of 32.45 μm with large variation (SD: ± 23.55 μm, Fig. 1G), which was significantly larger than that of retinal PrP^Sc^ deposits in the OPL (p < 0.001, Mann-Whitney *U* test; p < 0.001, Welch’s unpaired *t*-test, Fig. 1I). The difference of standard deviations between the two groups was statistically significant (p < 0.001, Brown-Forsythe test).

As expected, extensive spongiform change and neuronal cell loss were evident in H&E-stained slides prepared from the sCJD decedent autopsy brain (Fig. 1B). In contrast, histologic findings of severe neurodegeneration were not observed in sCJD retinas. The nuclear lamina of the retina (RPE, ONL, INL, and GCL) were all intact and of similar thickness (a proxy for retinal neuronaldensity) in sCJD retinas compared to the normal control retinas (Fig. 1C and D). Similarly, the synaptic/axonal/dendritic lamina of the retina (NFL, IPL, and OPL) also appeared intact and of similar thickness in all sCJD retinas and normal control retinas, despite strong PrP^Sc^ deposition observed in the OPL (Fig. 1D, E, 1F).

Our findings confirm prior reports of retinal PrP^Sc^ deposition in sCJD cases (Head et al., 2003, 2005; Orru et al., 2018; Takao et al., 2018) as well as in mouse models (Striebel et al., 2021). As with these studies, we find the strongest PrP^Sc^ deposition in the OPL where discrete ovoid deposits of PrP^Sc^ result in a “beads-on-a-string” appearance along the horizontal length of the lamina. This pattern of PrP^Sc^ immunoreactivity is similar to the strong ovoid OPL PrP^Sc^ immunohistochemical labeling observed previously (Head et al., 2003; Takao et al., 2018). We did not observe the “fine-dot” PrP^Sc^ staining in the INL, ONL, GCL, and NFL specifically reported by Takao and colleagues in some of their CJD cases (Takao et al., 2018). Possible reasons for the differences between our study and this prior report could arise from differences in the antibodies used to detect PrP^Sc^, tissue preparation conditions (possible autoclaving of tissue in Takao and colleague’s study vs proteinase K treatment in our study), or sCJD subtypes in the different patient cohorts. Their study included a higher proportion of MM1 sCJD cases (9 out of 11) than in our patient group (2 out of 14), and their cohorts also included 3 familial and 1 iatrogenic CJD case.

Furthermore, we found that the greatest diameters of the PrP^Sc^ deposits in the OPL of the retina were significantly smaller and with reduced variability in size compared to deposits in the brain. The greatest diameters of PrP^Sc^ deposits were also previously measured in the brains of 11 patients with vCJD by Armstrong and colleagues (Armstrong et al., 2005). In their study, the mean greatest diameters of diffuse and florid-type PrP^Sc^ deposits ranged from 7.5 μm to 42.3 μm across 12 different brain regions and also showed a positively skewed distribution similar to the sizes and distribution observed with the PrP^Sc^ deposit measurements in our sCJD brain autopsy tissues. These findings further support that brain PrP^Sc^ deposits differ markedly in size and morphology compared to retinal deposits.

Our current study focused on PrP^Sc^ deposits in the OPL because these are consistently the strongest sites of deposition in the sCJD retina. In contrast to the highly variable morphology of PrP^Sc^ deposits in the sCJD brain, there is a much more uniform and stereotypic pattern of PrP^Sc^ deposition in the OPL of the retina. Although further ultrastructural investigations of the structures involved in the OPL PrP^Sc^ deposits are warranted, we hypothesize that PrP^Sc^ is accumulating at the synaptic ribbon where the presynaptic terminals of rod and cone photoreceptors from the ONL connect with the postsynaptic dendrites of bipolar and amacrine interneurons from the INL. Evidence of this PrP localization has recently been reported by Striebel et al. (2021), who identified PrP^Sc^ accumulation in ribbon synapses and cilia of photoreceptors in mouse models of prion disease. The presence of PrP^Sc^ deposits at retinal synaptic junctions could alter or disrupt this retinal circuitry, result in photoreceptor degeneration, and perhaps contribute to visual symptoms reported in sCJD.

Counterintuitively, the extensive spongiform change, neuronal loss, and gliosis seen in association with PrP^Sc^ deposition in sCJD brain tissues were not found in association with PrP^Sc^ deposition in the retina. The absence of retinal degeneration in sCJD could potentially explain the clinical observation that patients experience symptomatic visual disturbances rather than frank anopsia. Although the etiology is unknown and ultrastructural investigations are needed, this phenomenon of minimal change prion retinopathy observed in our sCJD cohort could be attributed to either a delayed time course of PrP^Sc^ deposition relative to that of the brain or may be related to the genotype. It is worth noting that brain PrP^Sc^ deposition is not always associated with vacuolation in some prion diseases, such as in fatal familial insomnia and sporadic fatal insomnia (Abu-Rumeileh et al., 2018; Halliday et al., 2014).

Our observation of minimal change prion retinopathy lends credence to a previous investigation by Head and colleagues who reported no evidence of spongiform change in the retina from two vCJD cases and one sCJD case (Head et al., 2003). Head and colleagues did, however, observe increased GFAP expression in the nerve fiber layer (NFL) in the retina of one of their CJD cases (Head et al., 2003). Additionally, NFL thinning, reduced RGC numbers and optic nerve gliosis and atrophy have been reported in other sCJD patient case reports (de Seze et al., 1998; Lesser et al., 1979; Roth et al., 1979; Sato et al., 1992; Tarkkanen and Haltia, 1980; Tsutsui et al., 1986). More recently, a small yet statistically significant thinning of the NFL was observed in the retinas of sCJD patients relative to normal controls through the use of premortem optical coherence tomography by Orru and colleagues (Orru et al., 2018). Altogether, these findings suggest that neurodegenerative sequelae observed in the inner retina structures (GCL and NFL) and the optic nerve during the course of sCJD, may be either a direct consequence of OPL PrP^Sc^ deposition, an indirect consequence of other sCJD related ocular stressors (e.g. accumulation of redox-active iron), or a combination of such contributing factors (Chaudhary et al., 2021).

Although retinal lamination was normal, and the thickness of retinal lamina appeared similar in our sCJD cases compared to controls, we cannot exclude gliosis or microglial changes as we did not have sufficient tissue sections to stain for GFAP or other markers. Another limitation of our study is that we did not quantify retinal neuron numbers in our samples and therefore, cannot exclude neuronal loss in our sCJD cases.

Minimal change prion retinopathy may be a pathology limited to human prion disease. Several animal studies found photoreceptor degeneration, but not RGC degeneration, in scrapie-infected mice (Kercher et al., 2007; West Greenlee et al., 2016), and spongiform retinal degeneration in transmissible mink encephalopathy-affected cattle (Smith et al., 2009). The study of Kercher and colleagues also found activated microglia associated with neurodegeneration in the mice retina (Kercher et al., 2007). Future studies to quantify photoreceptor numbers in human sCJD retina may better identify signs of neurodegeneration. Also, investigating the status of microglia and Muller glia in sCJD retina may provide insight into the cellular responses to PrP^Sc^ deposition in the eye.

One possible explanation for the resilience of human retina compared to the brain to PrP^Sc^ neuropathology is a recent report that found natural abundance of the β-cleavage product of PrP^C^, N2, in human ocular tissue in contrast to the predominant *α* cleavage of PrP^C^ in the brain producing N1 and C1 products (Chaudhary-Suman, Association for Research Vision Ophthalmology. 2021 annual meeting, poster). Several studies suggest that the N2 fragment has a neuroprotective, antioxidant effect (Haigh et al., 2015; Watt et al., 2005). Given the oxidative stress and inflammatory milieu previously observed in prion diseases (Brazier et al., 2006; Guentchev et al., 2000; Lee et al., 1999; Singh et al., 2020), the antioxidant effect of the N2 byproduct may play a role in inhibiting significant retinal degeneration and explain why retinal PrP^Sc^ deposits are not associated with significant spongiform change. Another possibility is that the retina may be affected later than the brain in sCJD and our observations might be attributed to early retinopathy – e.g. overt neurodegenerative retinopathy might only be observed in atypical sCJD cases of prolonged duration.

Overall, our study corroborates prior reports that PrP^Sc^ deposits in sCJD retinas. In particular, PrP^Sc^ preferentially accumulates in the OPL of the retina where they adopt a stereotypic “beads-on-a-string” pattern in the form of ovoid aggregates at periodic intervals along the horizontal axis of the lamina. Regardless of spongiform change, neuronal loss, and gliosis, this stereotypic pattern of PrP^Sc^ deposition in the OPL could potentially help guide the development of diagnostic strategies for sCJD using retinal imaging modalities, such as adaptive optics (Qin et al., 2020). Similar to how the detection of retinal beta-amyloid in Alzheimer’s disease has been proposed as a diagnostic biomarker, viewing the pattern of PrP^Sc^ deposition in the OPL of the retina could allow for non-invasive testing and earlier diagnosis of clinically suspected sCJD cases (Koronyo et al., 2017).

## Figures and Tables

**Table 1:** Patient demographic data and clinical features of pathology-confirmed sporadic creutzfeldt jakob cases^a^ and controls.

Patient No.	Gender	Age of onset	Disease duration (mo)	PRNP genotype at codon 129	PrP^Sc^ type	Clinical signs at onset	Visual Symptoms^b,c^
1	F	51	4	–	N/A	N/A	N/A
2	F	80	14	VV	N/A	N/A	N/A
3	F	59	3	MM	N/A	N/A	N/A
4	M	79	27	MV	2	Cognitive	No
5	F	63	2	MM	1	Language	Yes
6	F	55	24	MV	1–2	Behavior	No
7	M	60	1.5	MM	1	Cognitive/visual	Yes
8	M	60	20	MV	1–2	Visuospatial	Yes
9	M	69	15	MV	2	Behavior/memory	No
10	F	69	6	MV	1	Behavior/memory	No
11	F	56	10	MM	1–2	Language	No
12	F	57	4	MM	1–2	Motor	No
13	F	55	6	VV	2	Apraxia	Yes
14	F	69	10	MM	1–2	Cognitive/apraxia	Yes

Control					
Patient No.	Gender	Age	Cause of death	Brain pathology	Retinal pathology
**1**	M	82	Metastatic pancreatic adenocarcinoma	Gross: unremarkable Microscopic: unremarkable	Gross: unremarkable Microscopic: peripheral cystoid degeneration, rare drusen
**2**	M	66	Metastatic squamous cell carcinoma (SCC)	Gross: unremarkable Microscopic: unremarkable	Gross: unremarkable Microscopic: rare drusen^d^
**3**	M	69	Pulmonary MALT lymphoma	Gross: unremarkable Microscopic: N/A	Gross: unremarkable Microscopic: unremarkable
**4**	M	90	Bronchopneumonia	Alzheimer disease neuropathologic changes: A2, B3, C2^e^	Gross: unremarkable Microscopic: peripheral cystoid degeneration, numerous drusen
**5**	M	51	Esophageal SCC, liver cirrhosis	Gross: unremarkable Microscopic: N/A	Gross: unremarkable Microscopic: rare drusen
**6**	F	64	Probable Alzheimer disease	Gross: moderate diffuse cortical atrophy Microscopic: N/A	Gross: unremarkable Microscopic: unremarkable

This table reports 14 cases of patients diagnosed with brain autopsy-confirmed sCJD and 6 control patients (no clinical or brain autopsy evidence of sCJD). The first 3 sCJD patients represent the new cases studied and the remaining 11 cases were previously described in Orru et al. (2018). Patients with sCJD comprised 10 females and 4 males, with the age of onset ranging from 51 to 80 years of age (Mean: 63, Median: 60, SD: 8.96) and with the disease duration ranging from 1.5 to 27 months (Mean: 10.5, Median: 8, SD: 8.38). The PRNP genotypes of these patients were analyzed and classified into subtypes (14.29% VV, 35.71% MM, 42.86% MV and one case unspecified), and the PrP^Sc^ types are specified in the adjacent column. Clinical signs at onset including cognitive, language, behavior, memory, and visuospatial presentations, and visual symptoms^b,c^ developed during the disease course are reported in the remaining columns. The control group included 5 males and 1 female with the age ranging from 51 to 90 years old (Mean: 70.33, Median: 67.5, SD: 13.84). Following autopsies, each patient’s cause of death excluded sCJD, and the table reports the conditions they were suffering from. Brain and retinal pathology findings of each control autopsy are reported in the adjacent columns.

a All were classified as definitive sCJD upon pathology examination.

b Visual symptoms noted at first through last UCSF visit.

c Visual Symptoms included transient monocular vision loss, blurred vision, double vision and visuospatial dysfunctions.

d Drusen were highlighted with PAS stain.

e A: Aβ/amyloid plaque score (Thal phases), B: Neurofibrillary tangle score (Braak stage), C:Neuritic plaque score (CERAD); N/A: not available.

## Abbreviations

CJD: Creutzfeldt-Jakob disease
sCJD: sporadic Creutzfeldt-Jakob Disease
vCJD: variant Creutzfeldt-Jakob Disease
PrP^C^: Cellular Prion Protein
PrP^Sc^: prion or Scrapie isoform of the Prion Protein
Mab: Monoclonal Antibody
TSE: Transmissible Spongiform Encephalopathy
PrP^res^: Proteinase K resistant Prion Protein
IHC: Immunohistochemistry
IPL: Inner Plexiform Layer
OPL: Outer Plexiform Layer
RPE: Retinal Pigment Epithelium
ONL: Outer Nuclear Layer
INL: Inner Nuclear Layer
RGC: Retinal Ganglion Cell
GCL: Ganglion Cell Layer
NFL: Nerve Fiber Layer
